# The role of indirect genetic effects in the evolution of interacting reproductive behaviors in the burying beetle, *Nicrophorus vespilloides*

**DOI:** 10.1002/ece3.4731

**Published:** 2019-01-18

**Authors:** Mauricio J. Carter, Alastair J. Wilson, Allen J. Moore, Nick J. Royle

**Affiliations:** ^1^ Centre for Ecology and Conservation University of Exeter Penryn UK; ^2^ Present address: Departamento de Ecología y Biodiversidad, Facultad de Ciencias de la Vida Universidad Andrés Bello República 440 Santiago Chile; ^3^ Present address: Department of Entomology College of Agricultural and Environmental Sciences University of Georgia Athens GA 30602‐7503 USA

**Keywords:** indirect genetic effect, mating, mating system, resource defense, social plasticity

## Abstract

Social interactions can give rise to indirect genetic effects (IGEs), which occur when genes expressed in one individual affect the phenotype of another individual. The evolutionary dynamics of traits can be altered when there are IGEs. Sex often involves indirect effects arising from first‐order (current) or second‐order (prior) social interactions, yet IGEs are infrequently quantified for reproductive behaviors. Here, we use experimental populations of burying beetles that have experienced bidirectional selection on mating rate to test for social plasticity and IGEs associated with focal males mating with a female either without (first‐order effect) or with (second‐order effect) prior exposure to a competitor, and resource defense behavior (first‐order effect). Additive IGEs were detected for mating rate arising from (first‐order) interactions with females. For resource defense behavior, a standard variance partitioning analysis provided no evidence of additive genetic variance—either direct or indirect. However, behavior was predicted by focal size relative to that of the competitor, and size is also heritable. Assuming that behavior is causally dependent on relative size, this implies that both DGEs and IGEs do occur (and may potentially interact). The relative contribution of IGEs may differ among social behaviors related to mating which has consequences for the evolutionary trajectories of multivariate traits.

## 1 INTRODUCTION

The social environment is arguably the most dynamic and fluctuating environmental component organisms encounter and respond to (Royle, Russell, & Wilson, 2014; Taborsky & Oliveira, 2012). This is because in social interactions, the “environment” consists of other interacting individuals expressing phenotypes that are also subject to evolution (Moore, Brodie, & Wolf, 1997). These social environments differ from nonsocial environments such as temperature and food availability not just in their dynamism but also in their heritable properties. When the phenotype of a focal individual is affected by genes being expressed in another individual with whom they are or have been interacting, for example, when individuals respond to the behavior of another individual by changing their own behavior (i.e., social plasticity; Royle et al., 2014; Taborsky & Oliveira, 2012), these effects are known as indirect genetic effects (IGEs; McGlothlin, Moore, Wolf, & Brodie, 2010; Moore et al., 1997; Wilson, 2014). In contrast, direct genetic effects (DGEs) reflect the effect of the focal genotype on the expression of traits in the focal phenotype.

The evolutionary dynamics of traits under selection can be profoundly altered when there are IGEs as well as DGEs, as focal and nonfocal genotypes both contribute and respond to the social environment (Bijma, 2014; McGlothlin et al., 2010; McGlothlin, Wolf, Brodie, & Moore, 2014; Moore et al., 1997). Understanding the evolution of socially induced traits (i.e., traits that arise as a result of social interactions) therefore requires understanding both DGEs and IGEs (Bijma, 2014; Bijma & Wade, 2008; Bijma, Muir, & Van Arendonk, 2007; McGlothlin et al., 2010, 2014; Moore et al., 1997). Social interactions in the context of reproduction will be closely tied to fitness so IGEs are expected to be especially important under sexual selection (Moore, Wolf, & Brodie, 1998; Wolf, Brodie, & Moore, 1999) and when there is parenting (Miller & Moore, 2007; Wolf et al., 1999; Wolf, Moore, & Brodie, 1997).

IGEs associated with male–female interactions have been examined in the context of the speed and duration of mating in *Drosophila* spp. (Bacigalupe, Crudgington, Slate, Moore, & Snook, 2008; Edward, Poissant, Wilson, & Chapman, 2014; Saltz, 2013; Tennant, Sonser, & Long, 2014), CHC profile (Chenoweth, Rundle, & Blows, 2010a; Petfield, Chenoweth, Rundle, & Blows, 2005), propensity to copulate and postcopulatory behavior in a flatworm (Marie‐Orleach et al., 2017), and time to spermatophore attachment in a cricket (Hall, Lailvaux, Blows, & Brooks, 2010). In addition, Clark, Begun, and Prout (1999) showed that the success of male *Drosophila melanogaster* in sperm competition depended on the genotype of the female mate. In the context of male–male social interactions during reproductive competition for females, IGEs for aggression have been evaluated in *D. melanogaster* (Bailey & Hoskins, 2014; Saltz, 2013) and *D. serrata *(Chenoweth et al., 2010a; Petfield et al., 2005). More generally, IGEs influencing social dominance have been examined in a variety of invertebrate and vertebrate systems (Bailey & Hoskins, 2014; Moore, Haynes, Preziosi, & Moore, 2002; Saltz, 2013; Sartori & Mantovani, 2013; Wilson, Morrissey, et al., 2011). Furthermore, although there have been numerous studies of maternal genetic effects (which are IGEs of maternal or paternal traits on offspring phenotype such as growth rate) in the context of parenting (McAdam, Garant, & Wilson, 2014) and studies of IGEs arising from postzygotic parent–offspring interactions (e.g., Head, Berry, Royle, & Moore, 2012; Lock, Smiseth, & Moore, 2004), we are not aware of any studies on behavior expressed during prezygotic interactions involved in mating and reproduction in species with parental care. This is an important omission because mating behaviors (such as mating rate) and parental care behaviors are expected to co‐evolve (Alonzo, 2010; Head, Hinde, Moore, & Royle, 2014; Kokko, Klug, & Jennions, 2012). As a result, IGEs that occur in the context of mating may have important co‐evolutionary consequences for the expression and evolution of parental care behaviors, as they can speed up or retard evolutionary processes (Bijma et al., 2007; Moore et al., 1997).

In many mating systems, there is a temporal structure to social interactions that influence mating behaviors; for example, there can be sequential bouts of sexual selection when male–male competition is followed by female mate choice (Hunt, Breuker, Sadowski, & Moore, 2009). In such cases, IGEs can be viewed as arising from either first‐ or second‐order social interactions (Saltz, 2013). In a first‐order effect, the behavior of a focal individual is influenced by the (genes of the) conspecific(s) they are currently interacting with. In the second‐order effect, the behavior of a focal individual is influenced by the (genes of) the conspecific(s) involved in interactions prior to the current interactions. So when considering mating behavior of focal males, first‐order IGEs could arise from interaction with the female he is mating with and second‐order IGEs, for example, as a result of his competitive interactions with rival males prior to mating. With the exception of Saltz (2013), quantification of second‐order IGEs, such as the effect of competition between males (male–male interactions) on mating behaviors (male–female social interactions), is lacking. Such second‐order interactions are important to consider because in groups of individuals, dyadic interactions can be affected by the behavior of other individuals in the group (Saltz, 2013) and because in the context of reproduction, male–male competition often determines behaviors involved in mating and parental care (Andersson, 1994; Hunt & Hosken, 2014; Klug, Heuschele, Jennions, & Kokko, 2010; Kokko et al., 2012). In the burying beetle *Nicrophorus vespilloides*, for example, when there is contest competition for breeding resources, winning males are typically larger (Hopwood, Moore, & Royle, 2013; Hopwood, Moore, & Royle, 2014; Lee, Head, Carter, & Royle, 2014; Otronen 1988) and achieve higher mating success but provide less care than when contest competition does not occur (Hopwood, Moore, Tregenza, & Royle, 2015; Royle & Hopwood, 2017). It is the combination of the sequential encounters that affect evolutionary outcomes (Hunt et al., 2009), which are influenced by both the form of selection acting on traits and the genetic architecture involved.

**Figure 1:**
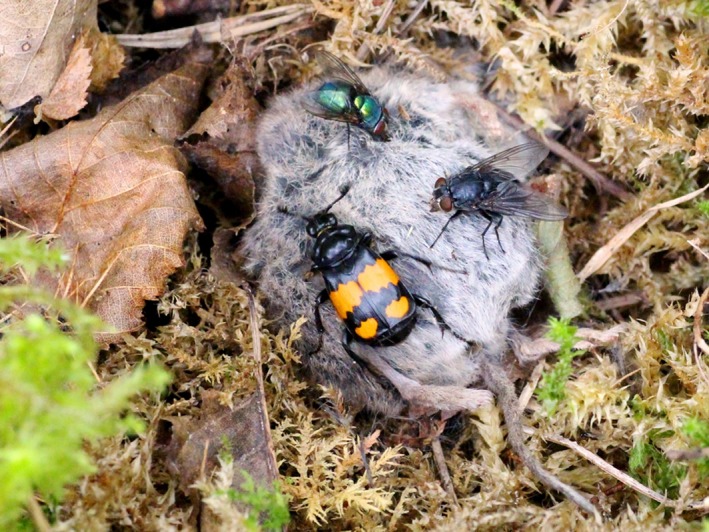
*Nicrophorus vespilloides* on carcass of common shrew *Sorex araneus* in the wild with interspecific competitors in attendance (blowflies, Calliphoridae). Photograph by Nick Royle

The primary focus of the current study was to elucidate sources of genetic variation underpinning socially plastic behaviors involved in reproduction in *N. vespilloides *(Figure 1). Previous work on *N. vespilloides* burying beetles shows that males respond to social interactions with rival males by changing their resource defense behavior (Carter, Head, Moore, & Royle, 2015) and their mating rate (Hopwood et al., 2015) and that, in the absence of social interactions with other males, there is underlying genetic variation in mating rate (mating rate responds to selection; Head et al., 2014). Although it is known that there is genetic variation for plasticity of resource defense behavior in response to social interactions with rival males (G × E; Carter et al., 2015), it is not known whether this is also the case for mating rate. Moreover, the source of the genetic variation underlying these traits (the genetic architecture) is unknown. In the current study, we therefore first tested for genetic variation underlying changes in male behavior in response to social interactions with rival males (G × E) and then quantified IGEs and DGEs on focal male reproductive behaviors arising from both male–male and male–female interactions. This provides insights into the genetic architecture and plasticity of traits involved in mating, and, therefore, how they might respond to selection and co‐evolve with parental care behaviors in *N. vespilloide*s, a species with extended, complex patterns of parental care (Eggert & Müller, 1997; Royle & Hopwood, 2017; Scott, 1998). We test for IGEs in two first‐order interactions, asking whether mating rate of focal males depends on female genotype (i.e., male–female interactions) and whether resource defense behavior depends on rival male genotype (i.e., male–male interactions). In addition, we examined a second‐order interaction, asking whether male mating rate is contingent on prior interaction with a rival and, if so, did the rival’s genotype have an effect on the outcome.

Previous work indicated that the genetic background of males, but not females, largely determined mating rate in *N. vespilloides* (Head et al., 2014) and that males respond plastically to competition from other males by changing their mating behavior (Hopwood et al., 2015). Accordingly, and because size is a strong predictor of success in contests in burying beetles (Hopwood et al., 2013; Hopwood et al., 2014; Lee et al., 2014; Otronen 1988), we predicted there would be first‐order IGEs associated with resource‐holding behaviors (male–male competitive interactions; e.g., Chenoweth, Rundle, & Blows, 2010b; Petfield et al., 2005; Wilson, Morrissey, et al., 2011) as well as second‐order IGEs of male–male interactions on mating rate. We expected first‐order IGEs arising from male–female interactions on mating rate, if present, to explain a relatively small amount of the genetic variation in mating rate compared to that of DGEs.

## 2 MATERIALS AND METHODS

### 2.1 Study organism

We studied the species of burying beetle *N. vespilloides *(Figure 1), which has complex socio‐sexual behaviors, including extensive, facultative parental care by one or both parents (Eggert & Müller, 1997; Royle & Hopwood, 2017; Scott, 1998). Reproductive behavior in burying beetles occurs both off and on the small vertebrate carcasses used for breeding (Eggert, 1992), and intrasexual competition (i.e., male–male and/or female–female interactions) for these resources is common (Otronen 1988). Intersexual competition for resources is not known to occur. Competition most frequently leads to the establishment of a dominant or “resource holder” male–female pair, who processes the carcass for breeding (Eggert & Müller, 1989; Pukowski, 1933). Beetles search for carcasses, and when more than one individual of the same sex are present at the same time, contests occur for access to the resources (the carcass, male beetles, and females), which determine the expression of alternative reproductive tactics (i.e., “resource holder” vs. “satellite”; Carter et al., 2015). Male success in contests is closely linked to body size (Hopwood et al., 2013; Hopwood et al., 2014; Lee et al., 2014; Otronen 1988) and “resource holder” males spend more time on the carcass, engaged in behaviors such as signaling (releasing pheromones), than “satellite” males, which spend more time off the carcass, looking for opportunities to sneak copulations with females (House, Hunt, & Moore, 2007; Müller, Eggert, & Dressel, 1990). Males mate frequently with females, especially in the presence of other males (Hopwood et al., 2015). This repeated mating is an effective form of paternity protection behavior (Head et al., 2014; Hopwood et al., 2015; House et al., 2008).

### 2.2 Experimental setup

We used beetles from existing selection lines for two, complementary, levels of analysis: between‐line analysis to test for social plasticity and G × E of focal traits, and within‐line analysis to quantify sources of genetic variation (DGEs and IGEs). All selection line beetles used in this experiment were collected from broad‐leaved woodland in Cornwall, UK, from the wild in July 2010. The focal males in our experiments were from generation F18 of four lines experimentally selected for high (two replicates) or low (two replicates) repeated mating rates (for details of selection regime and maintenance of beetles, see Carter et al., 2015 and Head et al., 2014). We have previously shown that mating rate differs between beetles from high and low lines, indicating that there is genetic variance in these populations that allowed the selection response (Head et al., 2014). These lines therefore provide an ideal opportunity to explore between‐line social plasticity and G × E and within‐line sources of genetic variation (DGEs and/or IGEs) for mating rate and related behaviors.

Nonfocal males used in our experiments to generate the competitive social environment experienced by focal males, and females, were derived from an outbred stock population collected from the wild during the summer of 2012 and maintained in the laboratory for four generations at a population size of between 500 and 1,000 individuals. Stock population beetles were therefore not known relatives of focal (selection line) males. We used a full‐sib design to provide pedigree information for all focals (selection line), and for competitor males and females (stock), to facilitate estimation of sources of genetic variation (all full‐sibs were reared with their parents so estimates of IGEs and DGEs are potentially confounded by common environmental effects during the larval stage including parental effects, but were subsequently housed thereafter in individual containers (i.e., during pupation and as adults, a period of ~4–5 weeks, until used in experiments) so postlarval common environment effects were minimized. A total of 498 focal males from 60 families (15 families per replicate line), 231 nonfocal (stock) males from 45 families, and 498 (stock) females from 90 families were used for the experiment. All individuals used were sexually mature, and unmated, virgins aged 13–15 days posteclosion. Prior to behavioral observations, all beetles were kept in individual containers (clear plastic container: 7 × 7 × 4 cm) filled with 2 cm of moist soil (Head et al., 2014). Mass (to 0.0001 g using an Ohaus Explorer balance) and size (pronotum width; to 0.1 mm using dial calipers) of all beetles were measured prior to behavioral experiments. For behavioral observations, focal males were placed in a transparent plastic box (17 × 11 × 6 cm) containing 1 cm of moist soil and a 15–25 g freshly thawed mouse carcass.

### 2.3 Male competition treatment

We evaluated the consequences of male–male competition on two reproductive behaviors expressed in focal males: resource defense (with or without a rival male present, depending on treatment group) and mating rate (with just a female present). Focal males from each selection line were allocated to one of two premating treatment groups: 1. competitor male present (*N* = 231) or 2. competitor male absent (*N* = 267). The competitor‐absent treatment was not required in order to quantify IGEs but was necessary to verify that behaviors were socially plastic and to test for G × E. Behavioral observations were done in 13 blocks with approximately 40 focal males per block. Experimental boxes of beetles were kept in an incubator with controlled temperature (21 ± 1ºC) and photoperiod (18 Light–6 Dark), as per previous studies (Carter et al., 2015; Head et al., 2014). All focal males were added to the container with the breeding resource (mouse carcass) and left alone for 24 hr to establish residency. Behavioral observations on focal males in the treatment group without a competitor were conducted 48 hr after introduction of the male to the experimental container. In the “competitor‐present” treatment group, a nonfocal, stock male was added to the experimental container 24 hr after the focal male and the two males were allowed to acclimate and interact for a further 24 hr before focal male behavior was recorded (Carter et al., 2015). This protocol of staggering arrival of males at the carcass replicates male arrival at carcasses in the wild (Hopwood, Moore, Tregenza, & Royle, 2016). Competitor males were given a white mark on their pronotum using correction fluid to facilitate the identification of focal and nonfocal (competitor) males. Marking does not affect behavior (Hopwood et al., 2013).

To determine resource defense behavior, we measured the activity of males on a carcass both in the presence and in the absence of a competitor (Carter et al., 2015). Previous work has shown that male size is the most important determinant of contest outcomes over carcasses (Hopwood et al., 2013; Hopwood et al., 2014; Lee et al., 2014; Otronen 1988) so competitor males were randomly drawn from one of the 45 families in the stock population, which did not differ in mean or variation in size from the population of focal, selection line, males (focal male pronotum width = 5.01 mm ± 0.05 (95% CI), competitor male pronotum width = 5.03 mm ± 0.05 (95%CI); *F*_1,460_ = 0.43, *p* = 0.51). We measured the resource defense behavior of males during the last 4 hr of light, when the activity of this species peaks (Eggert, 1992). During this time, male burying beetles perform conspicuous behaviors, either signaling (emitting pheromones) to attract females or other activity (eating, self‐grooming, or walking), both on and around the reproductive resource (Pukowski, 1933). We used scan sampling every 10 min for 4 hr (i.e., *N* = 24 for each focal individual) between 1,400 and 1800 hrs and recorded the amount of time males spent on these behaviors (Carter et al., 2015). Male position was recorded (on carcass, off carcass, or not visible) and, if visible, what behaviors they were engaged in (signaling, eating, self‐grooming, and walking). The amount of time spent signaling or performing other behaviors (eating, self‐grooming, or walking) on the carcass during these scans (carcass activity; CA) was our measure of resource defense (Carter et al., 2015).

### 2.4 Mating behavior

Twenty‐four hours following the male competition treatment outlined above, each focal male was removed from the experimental boxes and allowed to mate with a female, randomly drawn from one of the 90 families in the stock population, and repeated mating rate was quantified. Mating trials were conducted in a Petri dish (8.5 cm diameter) lined with filter paper, and male mating behavior was recorded for 1 hr following male introduction to the female (Head et al., 2014). Mating rate (MR) was defined as the number of times a male successfully inserted his aedeagus into the female in 1 hr (see Head et al., 2014).

### 2.5 Data analysis

Data were analyzed using a series of univariate and multivariate mixed models fitted by restricted maximum likelihood and implemented in ASReml version 3.0 (Gilmour, Gogel, Cullis, & Thompson, 2009). Throughout we assume Gaussian error structures. Visual inspection of model residuals suggested this was reasonable. To test the significance of the fixed effects, we used Wald *F* tests. The significance of random effect (co)variance terms was tested using log‐likelihood ratio tests comparing models with different random effects structures. For tests of single variance components, the test statistic, calculated as twice the difference in model log‐likelihood, is assumed to have a distribution corresponding to a 50:50 mix of chi‐square (*χ* ^2^) distributions having 0 and 1 degree of freedom, respectively (Self & Liang, 1987). For testing covariances, and comparing between models differing at more than one random term, the appropriate mix of chi‐square distributions is not as simply defined so we pragmatically (and conservatively) assume a chi‐square distribution with *n* degrees of freedom where *n* is the number of additional parameters in the more complex model. Response variables were mean standardized prior to analysis.

**Figure 2:**
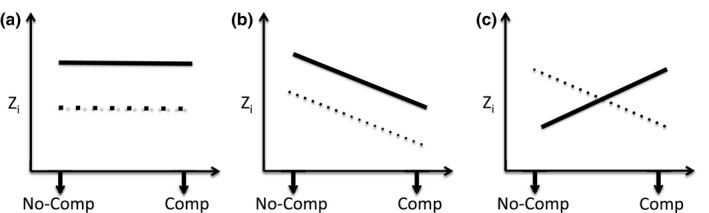
Potential responses of beetles from regimes selected for high (unbroken line) or low (dotted line) repeated mating rate to a change in social environment (absence (No‐comp) or presence (Comp) of male–male competition) for response variables (*Z*). (a) Illustrates the scenario where there is a difference in the mean values of traits between individuals from different selection regimes but no plasticity across social environments. (b) the selection regimes differ in mean values and there is plasticity but no G × E (selection regime × environment interaction). In contrast, (c) shows a significant G × E

We first modeled carcass activity (CA), for focal male *i* as follows:

$${\mathrm{CA}}_{i}\;=\;+\;\mathrm{Line}\;+\;{\mathrm{Replicate}}_{\mathrm{Line}}\;+\;\mathrm{Context}\;+\;\mathrm{Line}.Context\;+\;a_{i}\;+\;m_{j(\mathrm{context}\;=\;2)}\;+\;e_{ij.\mathrm{context}}$$

where *μ* is the mean, Line is a two‐level factor denoting the selection regime of focal origin (high and low selection on MR), Replicate_Line_ is a two‐level factor (replicate one or two) nested within each line type, and Context is a two‐level factor denoting competitive environment (1 = no competitor, 2 = competitor *j* present). A significant effect of Line would indicate the presence of between‐line (direct) genetic variance. Since selection was on MR, this would indicate a correlated response by CA. A significant effect of Context implies a plastic response (on average) by focal males to the presence of a rival. The Line.Context interaction term is included to test for between‐line G × E (see Figure 2). The Replicate_Line_ effect was included simply to account for any differences in phenotypic mean between replicates within high and low selection regimes.

**Figure 3:**
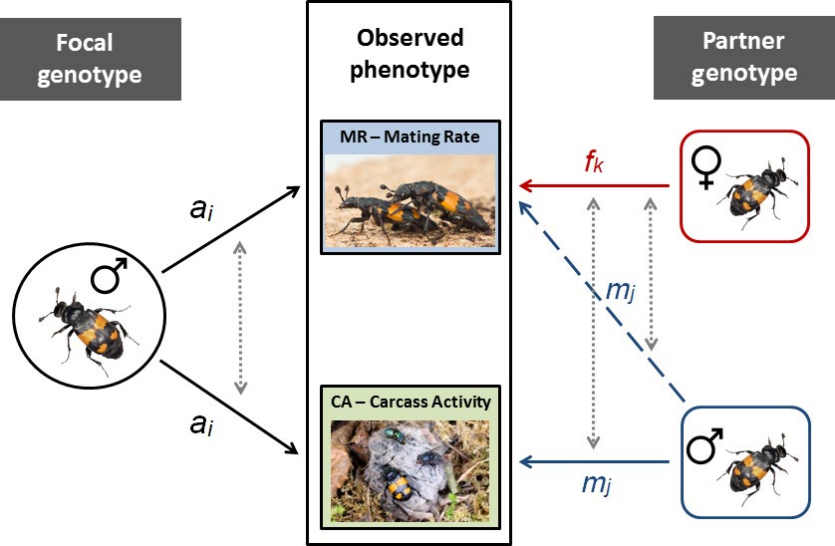
Variance and covariance components estimated in our experimental design. Mating rate (MR) and resource defense (CA) behaviors of focal males maybe influenced by direct genetic effects (*a_i_*) and/or indirect genetic effects as a result of social interactions with both rival, competitor males (*m_j_* with first‐order effects indicated by the solid arrow and second‐order effects indicated by the dashed‐line arrow) and females (*f_k_*—a first‐order effect). Covariance relationships estimated in our models are indicated by the dotted‐line arrows. See main text for more details. Photographs by Paul Hopwood, Jena Johnson, and Nick Royle

Our principal focus was on among‐individual genetic variance so we modeled random additive effects of focal (*a_i_*, the DGE) and, where Context = 2, a competitor male (*m_j_*, the first‐order IGE) genotype. These were assumed to be drawn from normal distributions with means of zero and variances to be estimated using pedigree data in a standard animal model extended to include IGEs (Wilson, Gelin, Perron, & Réale, 2009). Note however that the pedigree structure does not span the focal males and competitor male categories so DGE‐IGE covariances were not modeled (See Figure 3 for illustration of (co)variance terms estimated). Residuals (*e_ij_*) were similarly assumed to be normally distributed and uncorrelated across observations. We elected to model heterogeneous residual variance across the two contexts as otherwise any increase in total variance in the presence of a competitor may cause upward bias in the magnitude of IGE variance. This was done by fitting separate context‐specific residual variances *V_R_*_(context = 1)_ and *V_R_*_(context = 2)_.

We then similarly modeled focal male mating rate (MR) as follows:

$${\text{MR}}_{i}\;=\;\mu \;+\;\text{Line}\;+\;{\text{Replicate}}_{\text{Line}}\;+\;\text{Context}\;+\;\text{Line}\text{.Context}\;+\;a_{i}\;+\;m_{j\text{(context}\;=\;2)}\;+\;f_{k}\;+\;e_{ijk.\text{context}}$$

where *f_k_* is the first‐order IGE of female genotype on focal behavior and all other terms are as described above (but *m_j_* is now a second‐order IGE). Heterogeneous residual variance was again modeled. Covariance between the first‐order and second‐order IGEs is, in principle, estimable from our data structure since competitor males and females were drawn from the same stock population with known pedigree while DGE‐IGE covariances were again not estimable (see Figure 3).

The models above follow a “variance partitioning” framework for estimating IGEs in which the specific traits expressed by competitor males and/or partner females that influence focal male behavior are unknown. However, given prior work indicating that body size is a key mediator of male–male competition in burying beetles that is likely to be heritable, we elected to refit models of both traits but with an additional fixed effect of either (a) absolute opponent size (in Context 2 only) measured as pronotum width or (b) relative size (in Context 2 only) measured as the focal male's pronotum width divided by that of his opponent. For MR, we repeated these steps but using the pronotum width of the partner female in place of the opponent male to similarly test the possibility that IGEs from the female are size‐mediated. Since a causal dependence of focal behavior on the size of the male opponent and/or female partner implies the existence of IGEs if, and only if, size is heritable, we fitted an additional animal model where:

$$\mathrm{Pronotum}\;{\mathrm{width}}_{i}\;=\;\mu \;+\;\mathrm{Line}\;+\;{\mathrm{Replicate}}_{\mathrm{Line}}\;+\;{\mathrm{sex}}_{(\mathrm{Line}\;=\;stock)}\;+\;a_{i}\;+\;e_{i}$$

Note that in this analysis “Line” is a three‐level factor since stock individuals are included as focals in addition to individuals from high and low selection lines. Stock individuals with measured size included both males and females so a fixed effect of sex (two‐level factor) was also included to account for dimorphism.

Finally, we used a bivariate animal model of CA and MR (with all effects specified as described in univariate formulations above, i.e., not including any effects of size) to estimate the phenotypic covariance between the traits and test for direct (focal male) and/or indirect genetic contributions to this. For ease of interpretation, covariance estimates were scaled to the corresponding correlations.

## 3 RESULTS

### 3.1 Resource defense behaviors (carcass activity)

**Figure 4:**
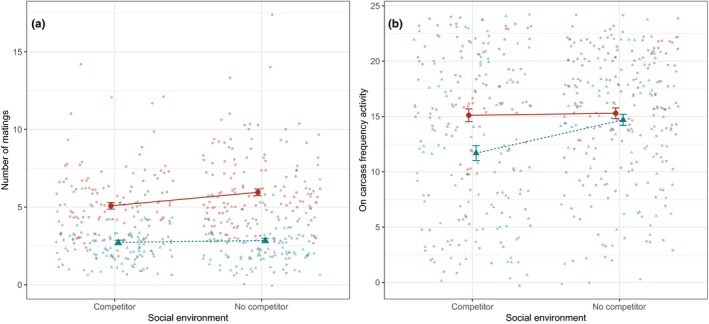
Predicted mean (± 1*SE*) male mating rate (MR) and resource defense (CA; amount of time spent active on the carcass) in relation to selection regime (Red circles = high line, Blue triangles = low line) and social environments experienced. The social environment (presence or absence of a rival, competitor male during resource defense prior to mating) is a second‐order effect for MR and a first‐order effect for CA; see main text for further details, including sample sizes

The univariate model of carcass activity (CA) indicated the presence of significant effects of Line, Context, and their interaction, but there was no significant effect of Replicate within lines (Table 1). Thus, we find evidence of a plastic response to competitor presence, as well as between‐line genetic variance and G × E. High‐line males spent more time on resource defense behavior (CA) than low‐line males but were largely unresponsive to a change in social context, whereas males from low lines had higher CA when they had not experienced competition from another male (Figure 4). Among individuals, we found no evidence for DGEs on CA with the estimate of focal male genetic variance bound to zero when constrained to lie in biologically meaningful parameter space (i.e., non‐negative variance; direct *h* ^2^ = 0.00, $\chi_{0,1}^{2}$ = 0.00, *p* = 0.500). (Note that relaxing this standard modeling constraint yields a small negative estimate of additive variance *V_A_* = −0.035 (0.950) which is not biologically meaningful). Nor was there evidence of significant IGEs on focal CA arising from the competitor male genotype in Context 2 ($\chi_{0,1}^{2}$ = 0.414, *p* = 0.26). Conditional on fixed effects, *m* ^2^ the proportion of variance explained by first‐order IGEs was low, with an estimate of *m* ^2^ = 0.047 (0.084) in the presence of a male competitor. However, our use of heterogeneous residuals did reveal an apparent difference in the variance structure of CA across the two contexts, with more residual variance estimated in the competitor context (*V_R_*_(context = 1)_ = 0.814 [0.071], *V_R_*_(context = 2)_ = 1.07 [0.133]). Although comparison of the model to a reduced formulation with a single homogeneous residual variance assumed suggests the inflation of residual variance in the presence of a competitor is marginally nonsignificant ($\chi_{\;1}^{2}$ = 3.002, *p* = 0.082), an increase in overall phenotypic variance (conditional on fixed effects) in Context 2 relative to 1 is statistically significant ($\chi_{\;1}^{2}$ = 6.904, *p* = 0.009). We thus suggest that focal CA has greater variance overall in the presence of a competitor.

**Table 1:** Parameter values and conditional *F* tests of fixed effects included in univariate models of resource defense (the amount of time spent active on the carcass [CA] and mating rate [MR]). Note that while coefficients (*SE*) are from the full model, *F* tests of Line and Context denote significance in the absence of any interaction terms

Model term	Factor level	Coefficient (*SE*)	*F*	*df*	*p*‐Value
CA					
*μ*		2.441 (0.096)	2,639.7	1, 171.3	<0.001
Line	Low	−0.071 (0.140)	9.22	1, 478.9	0.003
Replicate_Line_	2_High_	−0.046 (0.119)	0.36	2, 479.5	0.699
	2_Low_	−0.095 (0.126)			
Context	Competitor	−0.042 (0.126)	7.11	1, 66.0	0.010
Line.Context	Low.Competitor	−0.440 (0.179)	6.08	1, 448.4	0.015
MR					
*μ*		2.481 (0.113)	926.54	1, 49.0	<0.001
Line	Low	−1.397 (0.159)	116.29	1, 51.4	<0.001
Replicate_Line_	2_High_	−0.187 (0.141)	1.22	2, 50.2	0.303
	2_Low_	0.121 (0.146)			
Context	Competitor	−0.346 (0.097)	8.29	1, 440.7	0.004
Line.Context	Low.Competitor	0.302 (0.141)	4.62	1, 444.9	0.033

Refitting the model for CA with additional fixed effects to model size effects revealed that (a) absolute opponent size has no significant effect on focal male CA (coefficient = −0.050 [0.194] sdu/mm, *F*_1,189.4_ = 0.07, *p* = 0.791), but that (b) relative size does (coefficient = 3.653 [1.365], *F*_1,226.1_ = 7.16, *p* = 0.008). The positive sign in the latter result means that focal males increase CA when they are large relative to their opponent. The animal model of pronotum width yielded a moderate estimate of heritability for body size (conditional on fixed effects as described above) that was statistically significant (*h* ^2^ = 0.314 (0.057), $\chi_{0,1}^{2}$ = 72.5, *p* < 0.001). Thus, despite the fact that variance partitioning provides no evidence of (within‐line) additive DGEs or IGEs on resource defense (CA), if we accept that the statistical effect of relative size results from a causal relationship, this implies both genotypes do actually influence behavior through their joint determination of relative size.

### 3.2 Mating behaviors (mating rate)

For mating rate (MR), we also found evidence of a plastic response to competitor presence, as well as between‐line genetic variance and G x E (reflected by significant effects of Context, Line, and their interaction; Table 1). Predicted means show that high‐line males mated at a higher rate than low‐line males (as expected), and were more responsive, at the population level, to the change in social context than low‐line males. Thus, not only did high‐line males have a higher mating rate in the absence of premating competition with a rival than low‐line males, they also decreased MR if they had interacted with a rival before mating. In contrast, low‐line males did not change their behavior in response to a change in social context before mating (Figure 4). There was no significant difference between replicates within each line.

In contrast to CA, we also found evidence for both among‐individual direct genetic variance ($\chi_{0,1}^{2}$ = 13.9, *p* < 0.001) and first‐order IGEs arising from interactions with females ($\chi_{0,1}^{2}$ = 2.96, *p* = 0.043). However, there was no evidence of second‐order IGEs from the male competitors, with the estimate of male IGE variance bound to zero under standard modeling constraints (such that $\chi_{0,1}^{2}$ = 0.00, *p* = 0.500). Allowing the variance estimate to be less than zero yields an estimate of *V_M_* = −0.013 (−0.330) which we interpret as zero (as per the direct additive *V_A_* for CA described above).

The estimates of residual variance were quite similar in the two contexts (*V_R_*_(context = 1)_ = 0.512 [0.076], *V_R_*_(context = 2)_ = 0.445 [0.071]), and the heterogeneous formulation was not significantly better than a model containing a single *V_R_* ($\chi_{1}^{2}$ = 0.678, *p* = 0.412). Nor is there evidence for a significant difference in total phenotypic variance (conditional on fixed effects) across contexts ($\chi_{1}^{2}$ = 1.738, *p* = 0.187). Nevertheless, as a consequence of the heterogeneous residual structure fitted, the ratios of direct additive and first‐order indirect (female) genetic variances to phenotypic variance differ very slightly between contexts. Estimates of the direct heritability are thus $h_{\text{context}\;\;\text{=}\;\;\text{1}}^{2}$ = 0.211 (0.078) and $h_{\text{context}\;\;\text{=}\;\;\text{2}}^{2}$ = 0.233 (0.084). The first‐order (female) IGEs explained less variance overall, with estimates of $f_{\text{context}\;\;\text{=}\;\;\text{1}}^{2}$ = 0.074 (0.053) and $f_{\text{context}\;\;\text{=}\;\;\text{2}}^{2}$ = 0.082 (0.059). Note that with the variance explained by the second‐order (male) IGE bound to zero, we elected to drop the covariance between *m_j(context __= 2__)_* and* f_k_* in our model of MR as it was not possible to obtain model convergence from our data with this term parameter included and the covariance matrix of IGE constrained to be positive definite.

For MR, we found no evidence that focal behavior was mediated by either male opponent size (in Context 2) or female mating partner size. Thus, when added separately to our model, we found nonsignificant effects of both (a) absolute opponent size (coefficient = 0.013 [0.143] sdu/mm, *F*_1,240.1_ = 0.01, *p* = 0.920) and (b) focal size relative to opponent (coefficient = −0.806 [1.022], *F*_1,231.8_ = 0.62, *p* = 0.430) on MR. Similarly, we found no support for an effect of absolute female size on focal MR (coefficient = −0.018 [0.088] sdu.mm^−1^, *F*_1,470.0_ = 0.04, *p* = 0.829). Nor did focal male size relative to that of the female explain significant variance (coefficient = −0.054 [0.310], *F*_1,465.8_ = 0.03, *p* = 0.856).

### 3.3 Phenotypic covariance between CA and MR

Given the lack of detectable *h* ^2^ for CA and m^2^ for MR, the bivariate modeling added little further insight (full results not shown). The only estimable cross‐trait component of phenotypic covariance was the residual covariance (note we were unable to specify the bivariate model with each trait also having context‐specific *V_R_*), interpretable as arising from environmental effects not explicitly modeled. However, this scaled to a nonsignificant correlation close to zero (*r_R_* = −0.051 (0.054), $\chi_{1}^{2}$ = 0.886, *p* = 0.347). Thus, we find no support for phenotypic covariance between MR and CA conditional on the fixed and random effects as specified. This mirrors the absence of significant correlation in the observed data (Pearson’s correlation between MR and CA, *r*_496_ = 0.060, *p* = 0.181). We estimated the genetic correlation between male IGEs on CA and female IGEs on MR as *r* = 0.417 (0.594). We reiterate, however, that additive male IGEs on CA were not statistically significant (see earlier). In addition, dropping the covariance between IGEs in the bivariate model does not significantly reduce fit ($\chi_{1}^{2}$ = 0.296, *p* = 0.586). Taken together, this implies that the genetic correlation given above between IGEs (CA and MR) should be treated with caution.

## 4 DISCUSSION

The resource defense behavior (CA) and mating behavior (MR) of focal males were, on average, responsive to a change in social context (whether there was a rival male present or not during resource defense prior to mating); that is, both were socially plastic, with the extent dependent upon selection regime (among‐line G × E). However, the form of G × E differed between traits (Figure 4). Resource defense behavior (CA) did not differ between the lines when there were no competitive interactions with rivals, but in this experimental design when there were social interactions with competitors, low‐line males spent less time than high‐line males on resource defense (see also Carter et al., 2015). Low‐line males were therefore responsive to social context in CA, but high‐line males were not. In contrast, mating rate of low‐line males was lower than that of high‐line males regardless of social context, but especially when there were no social interactions with competitors. High‐line males were therefore responsive to social context in MR, but low‐line males were not. The direction of this effect of social environment on mating contrasts with previous studies in *N. vespilloides* such as Hopwood et al. (2015), where the presence of male competitors leads to an increase, not a decrease in mating rate. In the study by Hopwood et al. (2015), however, social interactions with competitor males occurred simultaneous to the mating interactions, whereas in the current study, competitive social interactions took place before mating.

At the within‐line level, significant genetic influences on behavior (and its plasticity) differed between traits. Mating rate was influenced by additive IGEs, arising from first‐order (male–female) but not second‐order (male–male) interactions, and DGEs. The former explained 7%–8% of trait variance and the latter 21%–23%. In contrast, our variance partitioning of models of resource defense behavior (without relative size included) provided no evidence for additive DGEs (explained ~0% of variance) or significant additive (first‐order) IGEs (~5% of variance explained). However, when relative size was included males’ socially plastic resource defense response to the presence of a rival male (change in CA) varied according to his size relative to the competitor. Furthermore, the dependence of behavior on relative size coupled with the nonzero heritability of pronotum width actually implies that IGEs are likely presented on CA. Although IGEs were not detected by variance partitioning, inclusion of partner traits as covariates should offer greater statistical power—provided the important traits are correctly identified. Here, if we accept that the statistical dependence of focal behavior on relative size reflects causality, then it follows that focal carcass activity depends jointly on the genotypes (with respect to body size) of both focal and competitor males. In fact, the use of a ratio to measure relative size implies not just additive DGEs and IGEs but also a multiplicative effect (i.e., G × G epistasis). However, refitting the model using focal size–competitor size as a proxy for relative size assumes additivity and results in a model fit that is not demonstrably different (not shown). Thus, we make no strong inferences here about nonadditivity. Nonetheless, it seems highly plausible that genes expressed in rival males that, for example, are associated with competitiveness (Carter et al., 2015) do contribute to genetic variation in the plasticity of focal male resource defense behavior.

Our analyses of relative size thus provide some evidence that IGEs, as well as DGEs, are involved in male–male competitive behaviors (resource defense; CA). However, we were unable to detect IGEs using variance partitioning alone, a result that contrasts somewhat with several other studies of dominance and aggressive behaviors. For example, Wilson et al. (2009) found evidence for IGEs for three of five aggression traits quantified in a study of agonistic behaviors during encounters between individuals of the same sex in deer mice *Peromyscus maniculatus*. Using a variance partitioning approach, this study also found positive correlations between direct and indirect effects which should facilitate rapid responses to (directional) selection. For a third trait, the correlation was negative, a situation predicted to constrain selection responses (Wilson et al., 2009; Wolf & Wade, 2001) and expected when the behavior is reflective of asymmetric dominance interactions. Positive selection on social status leads to correlated evolution of a more competitive environment, the spread of winning genotypes means that individuals are competing against more and more competitive opponents in each successive generation (Hadfield, Wilson, & Kruuk, 2011). This phenomenon of “evolutionary environmental deterioration” (Fisher, 1958; Frank & Slatkin, 1992) or the “treadmill of competition” (Wolf, 2003) has, for example, been shown in cockroaches *Nauphoeta cinerea* (Moore et al., 2002) and socorro isopods *Thermosphaeroma thermophilum *(Bleakley, Welter, McCauley‐Cole, Shuster, & Moore, 2013). In the limiting case, if contest outcome itself is viewed as a trait, IGEs necessarily impose an absolute constraint on phenotypic evolution: A genotype that directly predisposes to focal winning must indirectly predispose to losing when encountered in an opponent, and the mean rate of winning will always be 0.5 (Sartori & Mantovani, 2013; Wilson, 2014; Wilson, Boer, Arnott, & Grimmer, 2011).

Unlike the present experiment, in the studies highlighted above, competition‐associated IGEs were not specifically examined in the context of social interactions during reproductive opportunities between the sexes. However, studies of two Drosophila species have previously addressed this, testing for IGEs arising from intrasexual competition over access to members of the opposite sex. Using a trait‐based IGE modeling approach, Saltz (2013) showed that there were both first‐order (i.e., effects of opponent genotype) and second‐order (i.e., effects of the genotype of a third male) IGEs on focal male aggressive behavior in *D. melanogaster*. Interestingly, when the third male was from an aggressive lineage, subsequent interactions between focal and opponent were more escalated (Saltz, 2013). This means that the coefficients of interaction (denoted ψ) for partner on focal aggressiveness were positive for both first‐ and second‐order partner interactions. Analogous to finding positive IGE‐DGE correlations using variance partitioning, this should lead to accelerated phenotypic change in aggressiveness if under directional selection (Moore et al., 2002; Saltz, 2013; Wilson, 2014). However, in the Saltz (2013) study simple directional selection was not present, because focal mating success depended on the interaction of social context and genotype. Males with less aggressive genotypes had reduced mating success only when they were in more aggressive social environments (Saltz, 2013).

In contrast to findings in *D. melanogaster*, studies on *D. serrata *reported no evidence for IGEs from m‐m interactions. Petfield et al. (2005) combined manipulations of the social environment (group composition—alone, m‐m, or m‐f; type of contact possible—visual, touch, or mating) with a half‐sib mating design. Males rapidly changed CHC profile (an important cue for female mate choice) in response to interactions with females but not following interactions with rival males (Petfield et al., 2005). Subsequently, Chenoweth et al. (2010a) used selection lines of the same species to show this social plasticity in male CHC profile could itself evolve. This shows not only that IGEs from m‐f interactions were present, but that they can—and do—impact evolutionary trajectories of socially plastic traits (Chenoweth et al., 2010a).

In line with the conclusions of Chenoweth et al. (2010a), our analyses of *N. vespilloides* show that female genotype can influence male behavior, in this case mating rate. Thus, our variance partitioning analysis of mating rate supports the presence of first‐order (from the female) but not second‐order (from the previously encountered male rival) IGEs. The former result adds weight to the growing recognition of IGEs on mating behaviors (Bacigalupe et al., 2008; Edward et al., 2014; Hall et al., 2010; House et al., 2008; Tennant et al., 2014), including mate choice (Bailey & Zuk, 2012; Bailey & Macleod, 2014; Rebar & Rodriguez, 2013) and courtship provisioning (Teplitsky, Mills, Yarrall, & Merilä, 2010). The specific female trait(s) giving rise to indirect genetic variance in *N. vespilloides* is unknown but does not appear to be body size—either in an absolute sense or relative to that of the male.

What might be the consequences of these results for the evolution of mating rate in *N. vespilloides*? Our analysis shows that genetic variation for mating rate arises largely from direct genetic effects (i.e., variation among focal male genotypes). There is no detectable contribution from second‐order IGEs (i.e., the genotype of a specific rival male), although the presence of a rival (irrespective of his genotype) does lead to a plastic change in average focal mating rate. However, in addition to DGEs, genetic variance in mating rate also arises from IGEs derived from the female mating partner. A similar conclusion was also drawn by Head et al. (2014). In both studies, the evidence points to the male genotype being a more important determinant of mating rate than the female genotype with, for example, male DGEs explaining ~3× more variation in mating rate than female‐derived IGEs here. We acknowledge that it is possible that our direct genetic variance estimate could potentially be inflated (relative to wild‐type animals) by the use of selection line males (but stock females). However, line effects were included in the model to protect against this possibility. While the DGEs should facilitate a selection response, we note that our experimental design did not allow us to estimate the DGE‐IGE correlation (as the pedigree structure did not span test males and stock females). If this is negative, for instance because genes predisposing to high male mating rate also increase female resistance to males (Head et al., 2014; although as our results show, this is not related to body size of females), IGEs will constrain any directional selection response for mating rate. Regardless of the sign and subsequent dynamic, we can say that the presence of IGEs means we expect coevolution of male and female phenotypes. Furthermore, there was no evidence for second‐order IGEs associated with prior competition between rival males on mating rate. As a result, the co‐evolutionary effects on parental care traits found by Head et al. (2014) in the laboratory as a result of selection on mating rate in the absence of male rivals are likely to also hold in natural environments, even when there is prior male–male competition and female‐derived IGEs from mating.

Overall, our results are indicative of differences in plasticity and genetic architecture between the traits we measured. However, the picture is complicated. The among‐line G × E results showed that CA only differed between male beetles from low and high lines when they had experienced prior social interaction with a competitor, so although the main effect of line was significant, this was likely driven by the interaction between line and social context. This indicates that there was a correlated response by CA to selection on MR, albeit the change in trait mean was only apparent in the presence of a competitor. Logically, this divergence among lines could only have evolved given additive genetic variation for both MR and CA in the ancestral population. However, we found no evidence for DGEs underpinning CA in the among‐individual (within‐line) analyses. One possibility is that genetic variation is present, but is cryptic and only released by G × E and/or G × G interactions (Paaby & Rockman, 2014), with the latter including social epistasis (i.e., focal G × opponent G interactions). Regardless, the emergent picture for CA (nonadditive DGE‐IGE interaction mediated by size) contrasts with that for MR (presence of additive DGEs and IGEs, no mediation by size) and hints at genetical and functional separation of the behaviors. This fits with the mating system of *N. vespilloides* in which high levels of social uncertainty associated with the expression of alternative mating strategies are likely to select for behavioral plasticity (Royle & Hopwood, 2017). At the same time, co‐evolutionary feedback between mating and parental care behaviors is also known to be important (Alonzo, 2010; Head et al., 2014) and may ultimately be a more important determinant of the traits examined here than the effects of competition with rival males. For example, selection for an increase in male mating rate may lead to counter‐selection for increased male parental care if high mating rate imposes costs to female care that decrease male fitness (Head et al., 2014; Royle & Hopwood, 2017), independent of the social environment experienced during competitive interactions with rival males.

## 5 CONCLUSION

Our results show that changes in social behavior during reproduction can be complicated by a response (or lack of a response) to the genetical social environment. Although CA and MR both showed phenotypically plastic responses to changes in the social environment, the form and magnitude of the response differed, and there was evidence for a potential difference in genetic architecture. These traits may therefore have different evolutionary trajectories in response to selection. When there is competition between males for access to resources, directional selection on resource defense behavior (CA) would be unlikely to lead to a change in mean trait values, because variation in CA depends upon the genotypes of both the focal male and his rival (i.e., nonadditive effects), which will act to maintain variation. In contrast, directional selection on mating rate (MR) would be expected to lead to a positive response largely independent of the genetical social environment experienced (i.e., higher mating rates evolve even if there are IGEs associated with MR). Such differences among traits involved in reproduction could be common, highlighting the importance of quantifying IGEs as well as DGEs of traits to understand their evolution. Inferring evolution from phenotypic studies or fitness effects alone can be misleading with socially sensitive reproductive traits.

## CONFLICT OF INTEREST

None declared.

## AUTHOR CONTRIBUTIONS

N.J.R. and M.J.C. conceived the idea and designed the experiments. M.J.C. collected the data. M.J.C. and A.J.W. analyzed the data. All authors contributed to interpreting the data and writing the manuscript.

## DATA ACCESSIBLITY

Data available from the Dryad Digital Repository: https://doi.org/10.5061/dryad.9rk5f69.
